# Actualización sobre la resistencia antimicrobiana en instituciones de salud de nivel III y IV en Colombia entre enero del 2018 y diciembre del 2021

**DOI:** 10.7705/biomedica.7065

**Published:** 2023-12-01

**Authors:** Elsa De La Cadena, Christian J. Pallares, Juan Carlos García-Betancur, Jessica A. Porras, María Virginia Villegas

**Affiliations:** 1 Grupo de Investigación en Resistencia Antimicrobiana y Epidemiología Hospitalaria, Vicerrectoría de Investigaciones, Universidad El Bosque, Bogotá, D.C., Colombia Universidad El Bosque Universidad El Bosque Bogotá D.C Colombia; 2 Comité de Infecciones y Vigilancia Epidemiológica, Clínica Imbanaco, Grupo Quirónsalud, Cali, Colombia Clínica Imbanaco Grupo Quirónsalud Cali Colombia

**Keywords:** farmacorresistencia microbiana, monitoreo epidemiológico, Colombia, Drug resistance, microbial, epidemiological monitoring, Colombia

## Abstract

**Introducción.:**

El comportamiento de la resistencia antimicrobiana es fundamental en el mejoramiento y ajuste de los programas de optimización de uso de antimicrobianos, la implementación de las guías terapéuticas y las precauciones que limitan la transmisión cruzada de bacterias resistentes entre pacientes. Desde el inicio del 2020, la pandemia del SARS-CoV-2 desafió profundamente al sistema de salud y, según algunos reportes, aumentó las tasas de resistencia antimicrobiana.

**Objetivo.:**

Describir el comportamiento de la resistencia antimicrobiana en los microrganismos más frecuentes en veinte hospitales colombianos durante el periodo 2018-2021.

**Materiales y métodos.:**

Se trata de un estudio descriptivo basado en la información microbiológica reportada por veinte instituciones de salud de nivel III y IV, entre enero de 2018 y diciembre de 2021, en doce ciudades de Colombia, las cuales hacen parte del "Grupo para el estudio de la resistencia nosocomial en Colombia", liderado por la Universidad El Bosque. La identificación de género y especie de los microorganismos más frecuentes, junto con su perfil de resistencia frente a antibióticos marcadores, se determinaron mediante el análisis de los datos vía WHONET.

**Resultados.:**

En general, los 10 microorganismos más frecuentes analizados a lo largo de los 4 años no presentaron cambios estadísticamente significativos en sus perfiles de resistencia durante los cuatro años del periodo evaluado, de 2018 a 2021. En contraste, *Pseudomonas aeruginosa* aumentó su resistencia frente a piperacilinatazobactam y carbapenémicos, lo cual fue estadísticamente significativo.

**Conclusiones.:**

Los cambios en la resistencia antimicrobiana en estos años no han sido estadísticamente significativos, excepto para *P. aeruginosa,* bacteria que mostró un incremento en las tasas de resistencia a piperacilina-tazobactam y carbapenémicos.

## Abstract (en)

**Introduction.:**

Antimicrobial resistance surveillance is a fundamental tool for the development, improvement, and adjustment of antimicrobial stewardship programs, therapeutic guidelines, and universal precautions to limit the cross-transmission of resistant bacteria between patients. Since the beginning of 2020, the SARS-CoV-2 pandemic profoundly challenged the health system and, according to some reports, increased the rates of antimicrobial resistance.

**Objective.:**

To describe the behavior of antimicrobial resistance of the most frequent bacterial pathogens in twenty Colombian hospitals from January 2018 to December 2021.

**Materials and methods.:**

We conducted a descriptive study based on the microbiological information recorded from January 2018 to December 2021 in twenty levels III and IV health institutions in twelve Colombian cities. We identified the species of the ten most frequent bacteria along with their resistance profile to the antibiotic markers after analyzing the data through WHONET.

**Results.:**

We found no statistically significant changes in most pathogens' resistance profiles from January 2018 to December 2021. Only *Pseudomonas aeruginosa* had a statistically significant increase in its resistance profile, particularly to piperacillin/ tazobactam and carbapenems.

**Conclusions.:**

The changes in antimicrobial resistance in these four years were not statistically significant except for *P. aeruginosa* to piperacillin/tazobactam and carbapenems.

La vigilancia epidemiológica activa, en conjunto con actividades de prevención y control de infecciones, han demostrado ser medidas muy efectivas en la reducción y contención de la resistencia antimicrobiana ^1^^,^^2^. Por otro lado, los programas de optimización de antimicrobianos basados en datos de sensibilidad y resistencia microbianas son fundamentales para implementar las guías de antimicrobianos a nivel hospitalario, junto con la vigilancia y el seguimiento de los patrones de resistencia a nivel local, nacional, regional y global; además, permiten analizar el comportamiento de los agentes patógenos multirresistentes, así como ejercer una vigilancia microbiológica eficaz ^3^^,^^4^. Por todo lo anterior, el tener sistemas que garanticen una vigilancia de la resistencia antimicrobiana es esencial para implementar los programas de optimización de uso de antimicrobianos en cualquier hospital ^4^.

El uso indiscriminado de los antibióticos ha sido una de las causas principales de la diseminación y el aumento de la resistencia antimicrobiana a nivel mundial ^5^. Programas como el SENTRY *Antimicrobial Surveillance* y el *Study for Monitoring Antimicrobial Resistance Trends* (SMART) han reportado un aumento global en la resistencia antimicrobiana en los últimos años, especialmente para el grupo de agentes patógenos conocidos como ESKAPE *(Enterococcus faecium, Staphylococcus aureus, Klebsiella pneumoniae, Acinetobacter baumannii, Pseudomonas aeruginosa* y *Enterobacter* sp.) ^6^^,^^7^. Asimismo, Cantón *et al.* reportaron un aumento en la resistencia a las cefalosporinas de tercera generación en *Escherichia coli,* del 4,6 al 10,4 % entre el 2005 y el 2008, principalmente debido a la expansión del grupo clonal ST131, portador de β-lactamasas de espectro extendido (BLEE) CTX-M-15 ^8^. Para el caso de *K. pneumoniae,* también se encontró un aumento de la resistencia a dichas cefalosporinas: del 21,7 % en el periodo 1997-2000 al 36,1 % en el periodo 2013-2016 ^8^.

Por otro lado, se ha reportado un incremento en la resistencia a los carbapenémicos, especialmente en *K. pneumoniae,* la cual pasó del 0,6 en 1997-2000 al 2,9 % en 2013-2016, por carbapenemasas diferentes a KPC. Esta tasa es más notoria en Latinoamérica, ya que se ha reportado un incremento del 0,8 al 6,4 % durante el mismo periodo ^6^. Finalmente, para *P. aeruginosa,* se ha reportado un incremento de aislamientos multirresistentes, especialmente en Latinoamérica, donde pasó al 41,1 % en los últimos 20 años ^9^.

El aumento reciente de la resistencia antimicrobiana se ha asociado con la aparición de la COVID-19, especialmente en unidades de cuidados intensivos ^10^^,^^11^. En varios estudios se informó un aumento de la resistencia antimicrobiana durante los primeros 18 meses de la pandemia, siendo *A. baumannii* resistente a los carbapenémicos, S. *aureus* resistente a la meticilina (SARM), *Enterococcus* spp. resistente a la vancomicina, y *K. pneumoniae* y *P. aeruginosa*-MDR fueron los principales responsables de este incremento ^8^^,^^12^^,^^13^.

Durante la pandemia del SARS-CoV-2, las instituciones de salud fueron sometidas a una gran presión, demandándose grandes recursos del sistema de salud y obligando a muchas a reducir o, incluso, desarticular los planes o iniciativas de programas de optimización de uso de antimicrobianos ^14^. El personal de salud tuvo que ser reubicado para cubrir la emergencia, se aumentaron rápidamente las camas de cuidados intensivos, las áreas hospitalarias se dividieron en áreas COVID y áreas no-COVID, hubo escasez de personal entrenado e, igualmente, se reportó un aumento en el uso de antibióticos ^15^.

En paralelo con la infección por el SARS-CoV-2, se reportaron brotes por microorganismos multirresistentes, en gran parte, debido a la poca observancia de las normas de prevención y control de infecciones ^16^^-^^18^. Si bien los factores mencionados anteriormente contribuyeron al aumento de la resistencia antimicrobiana, también se observó que la disminución del número de procedimientos no esenciales, el distanciamiento social y el aumento en las medidas de limpieza y desinfección, así como un aumento general de la higiene de las manos, pudieron contribuir a su disminución ^14^.

Superada la fase crítica de la pandemia, es fundamental evaluar el comportamiento epidemiológico de la resistencia antimicrobiana en los principales agentes patógenos bacterianos y determinar si ocurrieron cambios importantes en sus patrones de resistencia a los antimicrobianos con respecto al periodo prepandémico. En este estudio, se describen la epidemiología bacteriana y el perfil de resistencia en instituciones de salud de Colombia pertenecientes al "Grupo para el estudio de la resistencia nosocomial en Colombia", durante los años 2018 a 2021.

## Materiales y métodos

### Diseño del estudio

Se trata de un estudio descriptivo realizado con la información de los aislamientos recopilados vía WHONET (versión 5.6) ^19^, en veinte instituciones de salud de nivel III y IV de doce ciudades colombianas, pertenecientes al "Grupo para el estudio de la resistencia nosocomial en Colombia" durante el periodo de enero de 2018 a diciembre de 2021.

Se hizo una búsqueda por frecuencia de aislamientos, tipos de muestras clínicas incidentes en estos aislamientos y perfiles de resistencia para los agentes patógenos más frecuentes, en particular, *E. coli, K. pneumoniae, P. aeruginosa* y *S. aureus,* frente a antibióticos seleccionados utilizados como marcadores de resistencia; estos últimos incluyeron ceftriaxona, cefotaxime, ceftazidime, cefepime, piperacilina-tazobactam, ertapenem, imipenem, meropenem, amikacina, ciprofloxacina, oxacilina y clindamicina.

Este análisis se llevó a cabo en todos los aislamientos reportados en las salas de hospitalización general y en unidades de cuidados intensivos de adultos.

### Población de estudio

Se seleccionaron veinte instituciones de salud de nivel III y IV en doce ciudades de Colombia que contaran con información completa de los cuatro años analizados (2018-2021). La información sobre todos los aislamientos de cada institución se obtuvo directamente de los sistemas automáticos de los laboratorios de microbiología, se utilizó para evaluar la sensibilidad antimicrobiana y fue recopilada por medio del *software* epidemiológico WHONET (versión 5.6) de la Organización Mundial de la Salud; posteriormente, se convirtió mediante el sistema de conversión de datos BacLink (versión 2.0) ^19^.

Estos datos fueron analizados por el Grupo de Resistencia Antimicrobiana y Epidemiologia Hospitalaria de la Universidad El Bosque, en Bogotá, Colombia. La información de los diferentes laboratorios de microbiología de las instituciones de salud se estandarizó mediante diccionarios para generar conglomerados según el tipo de muestra clínica. De igual manera, los puntos de corte para los diferentes antibióticos se establecieron según las directrices de la CLSI 2021 ^20^.

### Análisis de la información

Se tomó en cuenta el primer aislamiento de cada paciente de las salas de hospitalización general y de las unidades de cuidados intensivos, según la recomendación para realizar este tipo de análisis ^21^. Se determinaron los porcentajes de frecuencia de aislamientos, tipos de muestra más frecuentes y resistencia por año. Se utilizó la prueba estadística no paramétrica de Kruskal-Wallis para hacer comparaciones de grupos independientes y determinar diferencias en las proporciones de resistencias por año. Los valores de p menores de 0,05 se consideraron estadísticamente significativos.

## Resultados

### Aislamientos de salas de hospitalización general

Durante el periodo de análisis, se reportaron 61.184 aislamientos. Los diez microorganismos más frecuentes fueron, en orden descendente: *E. coli, K. pneumoniae, S. aureus, P. aeruginosa, Enterococcus faecalis, Proteus mirabilis, S. epidermidis, Enterobacter cloacae* complex, *Candida albicans* y *Serratia marcescens* (cuadro 1). Estos microorganismos representaron el 77 % de todos los aislamientos en los cuatro periodos.

**Cuadro 1:** Distribución y tendencia de la incidencia de los 10 microorganismos más frecuentes en salas generales en 20 instituciones de nivel III y IV durante el periodo 2018-2021 en Colombia (N=61.184)

Microorganismo	2018		2019		2020		2021	
	n	(%)	n	(%)	n	(%)	n	(%)
*Escherichia coli*	4084	26	4.568	28	3.749	26	3.814	26
*Klebsiella pneumoniae*	1.791	11	1932	12	1.829	13	1.845	13
*Staphylococcus aureus*	1.719	11	1.655	10	1.398	10	1.421	10
*Pseudomonas aeruginosa*	1.258	8	1.377	8	1.128	8	1.333	9
*Enterococcus faecalis*	831	5	836	5	780	5	711	5
*Proteus mirabilis*	637	4	824	5	693	5	706	5
*Staphylococcus epidermidis*	610	4	600	4	612	4	534	4
*Enterobacter cloacae* complex	476	3	446	3	500	3	492	3
*Candida albicans*	354	2	286	2	318	2	256	2
*Serratia marcescens*	310	2	310	2	284	2	326	2
Otros microorganismos	3.608	23	3.484	21	3.258	22	3.201	22
Total	15.678		16.318		14.549		14.639	

Durante el periodo 2018-2019, las muestras más frecuentes fueron las de orina, seguidas por las de piel y tejidos blandos, y en tercer lugar, las de sangre. Durante 2020 y 2021, la orina siguió siendo la muestra más frecuente, pero la sangre pasó a ocupar el segundo lugar y, la piel y los tejidos blandos, el tercero. En el cuadro 2 se muestran las muestras más frecuentes en los cuatro años del estudio. En las muestras de orina, *E. coli* se aisló en el 56 % de los casos, mientras que, para piel y tejidos blandos, *S. aureus* representó el 29 % de los aislamientos, seguido por *E. coli* con el 21 %. En sangre, *E. coli* representó el 22 % y S. *aureus* el 19 %. Llama la atención que *S. epidermidis* se aisló en el 16 % de las muestras de sangre, lo cual podría estar asociado con contaminación en la toma de la muestra.

**Cuadro 2:** Distribución por tipo de muestra más frecuente en salas de hospitalización general en 20 instituciones de nivel III y IV, durante el periodo 2018-2021 en Colombia (N=61.184)

Muestra	2018		2019		2020		2021	
	n	(%)	n	(%)	n	(%)	n	(%)
Orina	3.003	19,2	3.094	19,0	2.386	16,4	2.424	16,6
Piel y tejidos blandos	1.653	10,5	1.756	10,8	1.289	8,9	1.310	8,9
Sangre	1.415	9,0	1.490	9,1	1.700	11,7	1.744	11,9
Total	15.678		16.318		14.549		14.639	

**Figura 1:**
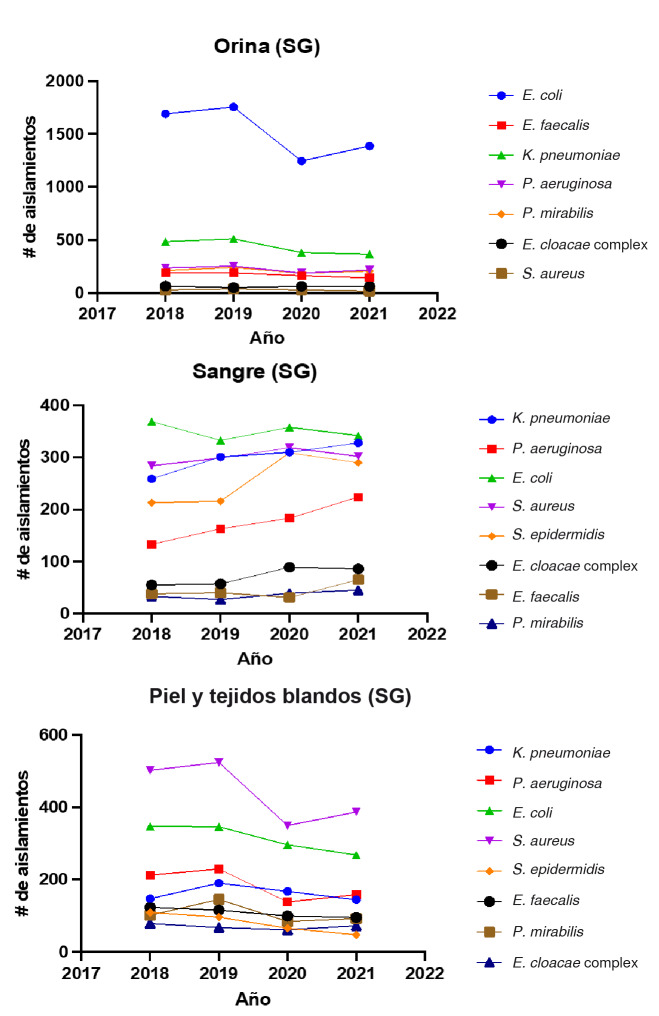
Tendencia del número de aislamientos bacterianos según los principales tipos de muestra aislados en salas de hospitalización general, durante los años 2018 a 2021

En la figura 1 se muestran las tendencias del número de aislamientos bacterianos por los principales tipos de muestra aislados en salas generales durante los años 2018 a 2021.

SG: salas de hospitalización general

### Aislamientos de la unidad de cuidados intensivos

Durante el periodo de análisis de los datos (2018-2021), se reportaron 32.523 aislamientos en la unidad de cuidados intensivos de adultos. Los diez microorganismos más frecuentes en los cuatro años fueron, en orden descendente: *E. coli, K. pneumoniae, S. aureus, P. aeruginosa, S. epidermidis, E. faecalis, C. albicans, P. mirabilis, E. cloacae* complex y S. *marcescens* (cuadro 3). Es de particular interés que se observó un incremento en el número de microorganismos aislados durante el periodo 2020-2021, especialmente de *K. pneumoniae,* que llegó a ser el primer microorganismo aislado en las unidades de cuidados intensivos por encima de *E. coli,* el cual ocupaba el primer lugar entre 2018 y 2019. En las unidades de cuidados intensivos de adultos, los diez microorganismos más frecuentemente aislados correspondieron al 74 % de todos los aislamientos en los cuatro periodos; las muestras más frecuentes durante el 2018 fueron, en orden descendente, sangre, secreción traqueal y orina. En el período 2020-2021, se incrementó considerablemente el número de cultivos positivos, y las secreciones traqueales pasaron a ser la muestra más frecuente (cuadro 4).

**Cuadro 3:** Distribución y tendencia de la incidencia de los 10 microorganismos más frecuentes en unidades de cuidados intensivos en 20 instituciones de nivel III y IV, durante el periodo 2018-021 en Colombia (N=32.523)

Microorganismo	2018		2019		2020		2021	
	n	(%)	n	(%)	n	(%)	n	(%)
*Escherichia coli*	989	17	1.193	17	1.097	13	1.320	12
*Klebsiella pneumoniae*	897	15	1.151	16	1.574	18	1.993	18
*Staphylococcus aureus*	616	10	684	10	720	8	1.025	9
*Pseudomonas aeruginosa*	500	8	656	9	792	9	1.193	11
*Staphylococcus epidermidis*	291	5	321	5	565	7	636	6
*Enterococcus faecalis*	255	4	268	4	376	4	495	5
*Candida albicans*	254	4	286	4	417	5	408	4
*Proteus mirabilis*	198	3	240	3	278	3	417	4
*Enterobacter cloacae* complex	186	3	171	2	306	4	377	3
*Serratia marcescens*	184	3	188	3	264	3	331	3
Otros microorganismos	1.554	26	1.841	26	2.257	26	2.759	25
Total	5.924		6.999		8.646		10.954	

**Cuadro 4:** Distribución por tipo de muestra más frecuente en unidades de cuidados intensivos en 20 instituciones de nivel III y IV, durante el periodo 20182021 en Colombia (N=32.523)

Muestra	2018		2019		2020		2021	
	n	(%)	n	(%)	n	(%)	n	(%)
Sangre	863	22,6	1.043	14,9	1.572	18,2	1.890	17,2
Traqueal	821	21,5	823	11,7	1.317	15,2	2.048	18,7
Orina	750	19,6	1.004	14,3	845	9,8	1.056	9,6
Total	5.924		6.999		8.646		10.954	

**Figura 2:**
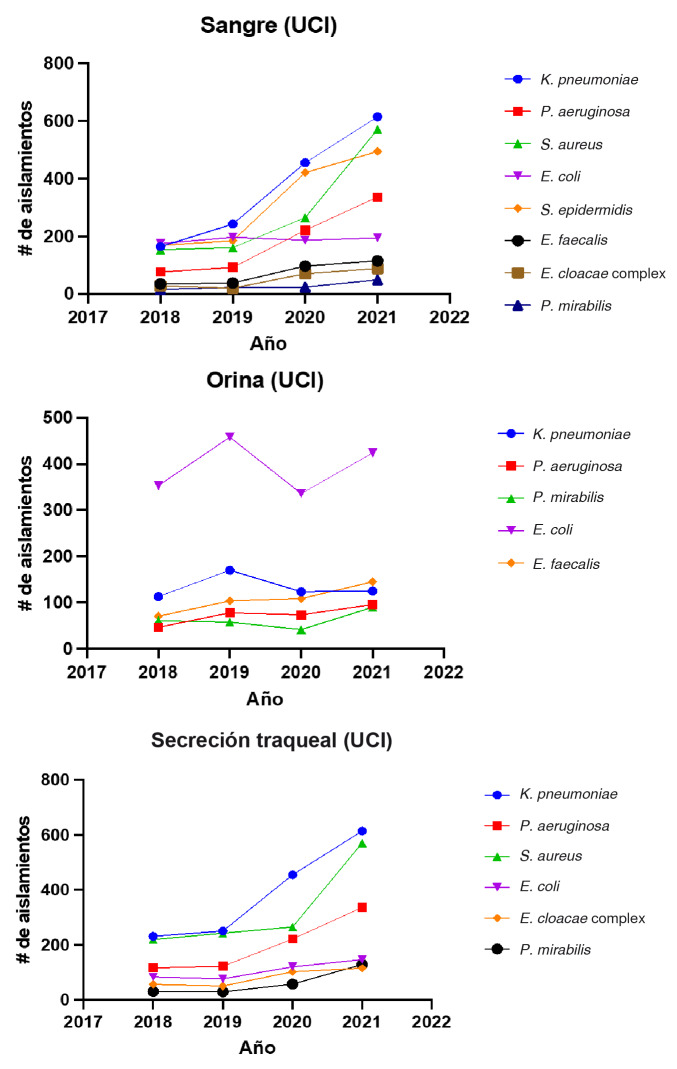
Tendencia del número de aislamientos bacterianos según los principales tipos de muestra aislados en unidades de cuidados intensivos adultos, durante los años 2018 a 2021

Los microorganismos frecuentes en sangre fueron, en orden descendente: *E. coli, K. pneumoniae, S. aureus* y *S. epidermidis;* mientras que, en orina, *E. coli* se aisló en más del 40 % de los cultivos. En secreciones traqueales, el microorganismo aislado con mayor frecuencia fue *K. pneumoniae,* seguido por *S. aureus* y *P. aeruginosa.* En la figura 2 se muestran las tendencias del número de aislamientos bacterianos según los principales tipos de muestra aislados en la unidad de cuidados intensivos durante los años 2018 a 2021.

UCI: unidad de cuidados intensivos

Es relevante que, en el año 2020, se observó un aumento en el número de aislamientos de *K. pneumoniae* en secreción traqueal: pasó de 231 aislamientos a 614 entre los años 2018 y 2020. Asimismo, se observó un aumento de *S. aureus* en el mismo tipo de muestra, pues en el 2021, su número fue de 571 aislamientos frente a 219 en el 2018. En sangre, *S. epidermidis* tuvo un incremento al pasar de 421 a 494 aislamientos en el 2020-2021 (figura 2).

### Resistencia de Escherichia coli y Klebsiella pneumoniae en salas de hospitalización general

**Figura 3:**
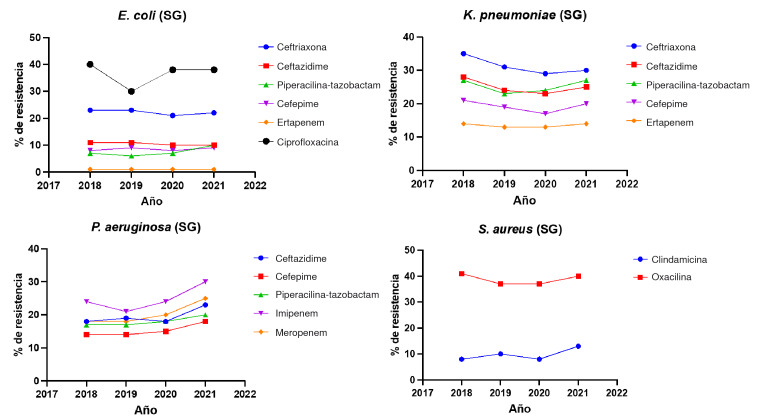
Tendencia del porcentaje de resistencia a antibióticos marcadores en salas de hospitalización general, durante el periodo 2018 a 2021

La resistencia de *E. coli* ante las cefalosporinas de tercera generación se mantuvo estable durante los cuatro años. La resistencia a la ceftriaxona fue del 22,2 % (21-23 %), como media, similar al comportamiento estable ante el ceftazidime, con una media del 10,5 % (10-11 %). En contraste, la mayor resistencia se presentó ante la ciprofloxacina, con el 36,5 % (30-40 %) (cuadro 5) (figura 3). Por otra parte, la resistencia de *K. pneumoniae* ante la ceftriaxona y el ceftazidime, disminuyó levemente entre el 2018 y el 2021, sin lograr la significancia estadística. En el caso de la ceftriaxona, fue del 31,2 % (29-35 %) y, con el ceftazidime, del 25 % (23-28 %) (cuadro 5) (figura 3).

**Cuadro 5:** Porcentaje de resistencia de los principales microorganismos aislados en salas de hospitalización general y unidades de cuidados intensivos durante 2018-2021

Microorganismo Antibiótico*	Salas de hospitalización general					Unidades de cuidados intensivos de adultos				
	Resistencia (%)				P**	Resistencia (%)				P**
	2018	2019	2020	2021		2018	2019	2020	2021	
*Escherichia coli*										
Ceftriaxona	23	23	21	22	NS	20	24	21	25	NS
Ceftazidime	11	11	10	10	NS	12	13	10	11	NS
Piperacilina-tazobactam	7	6	7	10	NS	8	9	11	14	NS
Cefepime	8	9	8	9	NS	9	9	8	10	NS
Ertapenem	1	1	1	1	NS	1	1	1	2	NS
Imipenem	1	1	1	1	NS	2	2	3	3	NS
Meropenem	1	1	1	1	NS	1	2	2	2	NS
Ciprofloxacina	40	30	38	38	NS	34	37	38	39	NS
Amikacina	0	0	1	1	NS	0	1	1	1	NS
*Klebsiella pneumoniae*										
Ceftriaxona	35	31	29	30	NS	32	35	27	27	NS
Ceftazidime	28	24	23	25	NS	25	27	23	22	NS
Piperacilina-tazobactam	27	23	24	27	NS	28	30	25	26	NS
Cefepime	21	19	17	20	NS	20	22	17	19	NS
Ertapenem	14	13	13	14	NS	16	21	17	15	NS
Imipenem	17	14	14	14	NS	18	22	20	18	NS
Meropenem	15	14	14	14	NS	17	22	19	18	NS
Amikacina	3	3	4	3	NS	0	6	3	2	NS
*Pseudomonas aeruginosa*										
Ceftazidime	18	19	18	23	NS	21	22	17	25	NS
Cefepime	14	14	15	18	NS	19	17	14	21	NS
Piperacilina-tazobactam	17	17	18	20	NS	21	22	18	25	<0,05
Imipenem	24	21	24	30	<0,05	27	30	26	34	<0,05
Meropenem	18	18	20	25	<0,05	25	25	20	28	<0,05
Amikacina	10	12	10	15	NS	10	14	10	14	NS
*Staphylococcus aureus*										
Clindamicina	8	10	8	13	NS	8	7	8	10	NS
Oxacilina	41	37	37	40	NS	28	28	26	26	NS

NS: no estadísticamente significativo

* La resistencia se determinó utilizando un grupo de antibióticos marcadores.

** Test de Kruskal-Wallis para comparación de grupos independientes

Para otros antibióticos como cefepime y piperacilinatazobactam, la resistencia en cepas de *E. coli* fue, como media, del 8,5 % y del 7,5 %, respectivamente y, aunque en el 2021 se observó un aumento en la resistencia de *E. coli* a estos antibióticos, no fue estadísticamente significativo. Para *K. pneumoniae,* la resistencia a estos dos antimicrobianos fue, como media, del 19,3 % y del 25,2 %, respectivamente (cuadro 5).

En el caso de los carbapenémicos, la resistencia de *E. coli* fue baja (alrededor del 1 %), en contraste con *K. pneumoniae,* cuya resistencia a ertapenem se consideró grande: 13 % (13-14 %). Sin embargo, al analizar el comportamiento de la resistencia a los carbapenémicos durante el periodo en estudio (2018-2021), tanto en *E. coli* como en *K. pneumoniae,* no se observaron cambios estadísticamente significativos (cuadro 5).

### Resistencia de Pseudomonas aeruginosa a TZP, cefepime y carbapenémicos en salas de hospitalización general

La resistencia de *P. aeruginosa* frente a ceftazidime y cefepime, se mantuvo estable (19 % y 14 %, respectivamente). En contraste, se observó un aumento estadísticamente significativo durante el 2021 con el imipenem y el meropenem, la cual pasó del 24 % al 30 % y del 18 % al 25 %, respectivamente (cuadro 5) (figura 3).

### Resistencia de Staphylococcus aureus a oxacilina y clindamicina en salas de hospitalización general

No se observaron cambios significativos en la resistencia de *S. aureus* ante la clindamicina y la oxacilina. La resistencia a la clindamicina fue, como media, del 10 % (8-13 %). La resistencia a la oxacilina se mantuvo estable, en el 39 % (37-41 %) en promedio (cuadro 5) (figura 3).

SG: salas de hospitalización general

**Figura 4:**
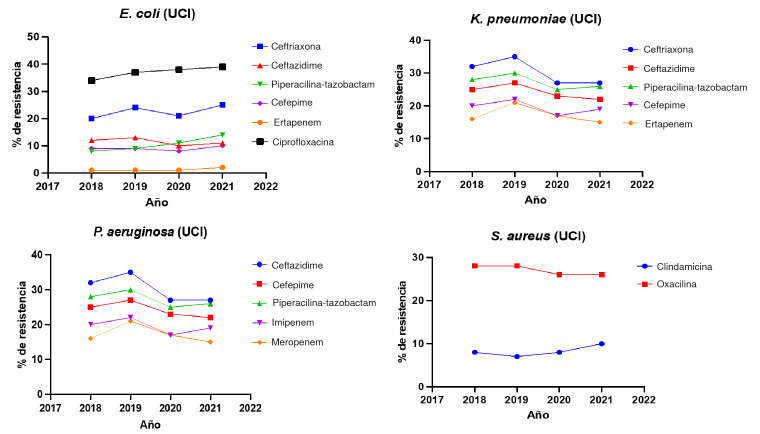
Tendencia del porcentaje de resistencia a antibióticos marcadores en las unidades de cuidados intensivos de adultos, durante el periodo 2018-2021

UCI: unidad de cuidados intensivos

### Resistencia de Escherichia coli y Klebsiella pneumoniae en cuidados intensivos

La resistencia de *E. coli* a la ceftriaxona fue estable, con una media del 22,5 % (20-25 %), similar al comportamiento estable de ceftazidime, con una media del 11,5 % (10-13 %) (cuadro 5). La resistencia a la ciprofloxacina en la unidad de cuidados intensivos para adultos aumentó en el período de estudio, aunque sin significancia estadística, fue del 37 % (34-39 %) (cuadro 5) (figura 4). Por otro lado, en *K. pneumoniae* la resistencia a ceftriaxona y ceftazidime disminuyó levemente entre el 2018 y el 2021, sin alcanzar la significancia estadística. La resistencia a la ceftriaxona en *K. pneumoniae* fue del 30,2 % (27-35 %) y, para ceftazidime, del 24,2 % (22-27 %) (cuadro 5) (figura 4).

Para el cefepime y la piperacilinatazobactam, la resistencia media de *E. coli* fue del 9 % y el 10,5 %, respectivamente; aunque en el año 2021 se observó un aumento de la resistencia a estos antibióticos, no fue estadísticamente significativo. Para *K. pneumoniae,* la resistencia a estos dos antimicrobianos tuvo una media del 19,5 % y el 27,2 %, respectivamente (cuadro 5).

Al igual que en las salas de hospitalización general, la resistencia de *E. coli* a los carbapenémicos en las salas de en cuidados intensivos fue baja, (alrededor del 1 %); en contraste, y similar a lo observado en las salas de hospitalización general, *K. pneumoniae* presentó gran resistencia al ertapenem, con el 18 % (15-21 %). Sin embargo, al analizar el comportamiento de la resistencia a los carbapenémicos tanto en *E. coli* como en *K. pneumoniae* durante el periodo en estudio (2018-2021), no se observaron cambios estadísticamente significativos (cuadro 5). Finalmente, la resistencia de *E. coli* frente a la ciprofloxacina fue la más grande entre todos los antibióticos tamizados; se situó en el 37 % (34-39 %), como media.

### Resistencia de Pseudomonas aeruginosa a piperacilina-tazobactam, cefepime y carbapenémicos en cuidados intensivos

En la unidad de cuidados intensivos de adultos, el ceftazidime y el cefepime tuvieron un comportamiento estable. Fue diferente con la piperacilina-tazobactam, que presentó un aumento significativo: la resistencia pasó de 21 % al 25 %. De igual manera, la resistencia de *P. aeruginosa* a imipenem y meropenem, aumentó significativamente: pasó de 27 % a 34 % y de 25 % a 28 %, respectivamente (cuadro 5) (figura 4).

### Resistencia de Staphylococcus aureus aureus a oxacilina y clindamicina en la unidad de cuidados intensivos de adultos

No se observaron cambios significativos en la resistencia de *S. aureus* a la clindamicina y la oxacilina. Fue similar con la clindamicina, con el 8 % (710 %) en promedio, en la unidad de cuidados intensivos disminuyó del 28 % en 2018 al 26 % en 2022, pero sin alcanzar significancia estadística (cuadro 5) (figura 4).

## Discusión

En este análisis se reportan el comportamiento de las frecuencias, el tipo de muestra más frecuente y el porcentaje de resistencia contra los antimicrobianos en los patógenos más frecuentes provenientes de pacientes hospitalizados en las salas de hospitalización general o en la unidad de cuidados intensivos de adultos, durante el periodo 2018-2021, incluyendo el inicio de la pandemia del SARS-CoV-2, la cual representó un inmenso desafío para el sistema de salud y los programas de optimización de uso de antimicrobianos ^22^.

De acuerdo con el análisis de estos datos, no se observaron cambios en el orden de frecuencia de los diez primeros microorganismos que se aislaron en el período de estudio, pero sí en el número de aislamientos, el cual disminuyó en las salas de hospitalización general, pero contrastó con un aumento de los aislamientos en la unidad de cuidados intensivos de adultos, particularmente durante el periodo 2020-2021. Esto podría explicarse por la suspensión de procedimientos no esenciales y otros factores, que disminuyeron la hospitalización en salas de hospitalización general durante los primeros meses de la pandemia, así como aumentó el número de adultos con diagnóstico de COVID-19 que ingresaron a la unidad de cuidados intensivos durante la emergencia ^14^.

Además, entre el 2018 y el 2019, *E. coli* fue la principal causa de infecciones en la unidad de cuidados intensivos, pero, entre el 2020 y el 2021, *K. pneumoniae* pasó al primer lugar ^23^. Estos hallazgos concuerdan con lo reportado en otros países, donde *K. pneumoniae* se convirtió en el principal agente etiológico en la unidad de cuidados intensivos de adultos debido al aumento de neumonías asociadas con el respirador en pacientes con la COVID-19 ^24^^,^^25^. Maes *et al.* reportaron que hasta el 80 % de los adultos con COVID-19 que ingresó a cuidados intensivos, requirió asistencia respiratoria mecánica invasiva, lo cual se sabe que es un factor de riesgo importante para desarrollar una neumonía asociada al respirador ^26^. En el presente estudio, en las veinte instituciones de salud, las muestras de origen respiratorio pasaron a ocupar el primer lugar en número de aislamientos durante el año 2021 y el 30 % de estas muestras mostraron la presencia *K. pneumoniae.*

Briceño *et al.* (2010) encontraron que las bacterias Gram negativas más frecuentes en la unidad de cuidados intensivos de adultos, durante el periodo 2006-2008 fueron, en orden descendente: *E. coli* (26 %), *K. pneumoniae* (9 %), *P. aeruginosa* (7 %), *E. cloacae* (3 %) y *A. baumannii* (1 %) ^27^. En en el presente análisis, se observó un cambio en la distribución de los microorganismos más frecuentes, tanto en salas de hospitalización general como en la unidad de cuidados intensivos de adultos.

En comparación, en un reporte del Instituto Nacional de Salud que incluyó datos del periodo 2012-2014, *A. baumannii* estaba entre los 10 principales microorganismos causantes de infecciones en la unidad de cuidados intensivos para adultos y *S. aureus* ocupaba un cuarto lugar ^23^. En los cuatro años que incluyen este informe, *S. aureus* pasó a ocupar el tercer lugar y *A. baumannii* no se ubicó dentro de los diez primeros microorganismos aislados en la misma unidad. La salida de *A. baumannii* de este grupo puede deberse a la implementación de programas de desinfección y limpieza más estricta en las instituciones participantes, ya que este agente patógeno es un gran colonizador en el ambiente hospitalario ^28^.

Con la vigilancia del Instituto Nacional de Salud del 2012 al 2014, se encontró que la resistencia de *E. coli* a las cefalosporinas de tercera generación era del 23,6 % en la unidad de cuidados intensivos de adultos y del 22,5 % en salas de hospitalización general. Además, en otro estudio sobre infecciones asociadas con la atención en salud, que incluía aislamientos recolectados del 2012 al 2018, se reportó una resistencia a dichas cefalosporinas en un rango de 14 al 31 %, con un promedio del 22,7 % en la unidad de cuidados intensivos y del 24 % en salas de hospitalización general ^23^^,^^29^. Comparativamente, el presente análisis mostró que la resistencia de *E. coli* a las cefalosporinas de tercera generación durante el periodo 2018-2021 se ubicó en el 22,2 % en salas de hospitalización general y en el 22,5 % en la unidad de cuidados intensivos de adultos, mientras que, para este mismo agente patógeno, la resistencia a carbapenémicos no sobrepasó el 1,5 % en ambos lugares.

Según los reportes del Instituto Nacional de Salud, *K. pneumoniae* mostró una resistencia a las cefalosporinas de tercera generación y de cuarta generación, del 38,3 % en salas de hospitalización general y de cerca del 30 % en la unidad de cuidados intensivos de adultos ^29^. En el presente estudio, se observó que la resistencia de *K. pneumoniae* a las de tercera generación, presentó valores del 30,2 % en salas de hospitalización general y del 31,2 % en la unidad de cuidados intensivos; esto fue similar a lo reportado previamente en las unidades de cuidados intensivos de adultos, pero con una menor tasa de resistencia en las salas de hospitalización general. Frente a la resistencia a carbapenémicos, el Instituto Nacional de Salud reportó el 14,9 % en salas de hospitalización general y el 15,6% en la unidad de cuidados intensivos de adultos, cifras que fueron ligeramente mayores en el presente estudio, del 16 y del 17,2 %, respectivamente.

Para el periodo 2012-2014, Ovalle *et al.* informaron tasas de resistencia de *P. aeruginosa* a carbapenémicos que oscilaban entre el 25,2 y el 28,7 % en las unidades de cuidados intensivos, y entre el 19,5 y el 23,3 %, en las salas de hospitalización general ^23^. En el presente estudio, se encontraron rangos superiores: la resistencia de *P. aeruginosa* fue del 29,2 % contra imipenem y del 24,5 % contra meropenem en las unidades de cuidados intensivos; y en las salas de hospitalización general, fue de 24,7 y 20,2 %, respectivamente, observándose un aumento estadísticamente significativo en ambos lugares.

En cuanto a *S. aureus,* su resistencia a la oxacilina reportada previamente se ubicó en el 38,5 % en las salas de hospitalización general y en el 31,3 % las unidades de cuidados intensivos de adultos ^23^; en el presente análisis fue ligeramente menor, sin significancia estadística: el 37,7 y el 27 %, respectivamente.

Por otro lado, si se comparan nuestros resultados con los del informe de vigilancia del Instituto Nacional de Salud por Whonet de la resistencia antimicrobiana en infecciones asociadas con la atención en salud, se encuentra que, mientras *E. coli* fue el microorganismo más frecuente en las unidades de cuidados intensivos en el 2019 ^30^, a partir del 2020 y del 2021, *K. pneumoniae* pasó a ocupar ese lugar ^31^^,^^32^, por encima de *E. coli,* similar a lo reportado en nuestro estudio. Sin embargo, durante el 2021, *P. aeruginosa* desplazó a *E. coli* del segundo puesto en las unidades de cuidado intensivo ^32^. En comparación, *E. coli* siguió siendo el segundo microorganismo más frecuente en las unidades de cuidados intensivos en nuestro estudio y *P. aeruginosa* se ubicó en el tercer puesto. Para el período 2020-2021, el Instituto Nacional de Salud reportó una tendencia al aumento en la resistencia a cefalosporinas de tercera generación en *E. coli* y *K. pneumoniae,* en ambos lugares ^31^^,^^32^.

En nuestro estudio, la resistencia de *E. coli* a las cefalosporinas de tercera generación se mantuvo estable durante los cuatro años del estudio, con una leve disminución para *K. pneumoniae.* En cuanto a la resistencia a los carbapenémicos, los datos del Instituto Nacional de Salud sobre el período 2020-2021 mostraron que se mantuvo estable en *E. coli* y *K. pneumoniae*^31^^,^^32^, igualmente, similar a los hallazgos en nuestro estudio. En general, las cifras de resistencia a las cefalosporinas de tercera generación fueron más altas en los reportes del Instituto Nacional de Salud, pero muy parecidas a las de nuestro estudio para los carbapenémicos ^30^^-^^32^. Los datos del Instituto Nacional de Salud sobre los años 2019-2021 incluyen información de 338 entidades de salud, en promedio; se observan porcentajes de resistencia más altos en algunas regiones, lo que podría elevar el promedio nacional y explicar las diferencias en los datos globales.

La pandemia de COVID-19 en Colombia se inició en marzo de 2020 y el 25 de agosto de 2022 se dio por terminada la emergencia sanitaria; en ese momento, se habían reportado 6'299.595 casos confirmados, 141.519 fallecidos y 6'122.457 recuperados de COVID-19 ^33^. En el consenso colombiano de atención, diagnóstico y manejo de la infección por SARS-CoV-2/COVID-19 en establecimientos de atención de la salud, se recomendó iniciar tratamiento antibiótico empírico en los pacientes con sospecha de neumonía bacteriana leve o moderada, acorde con guías nacionales o guías institucionales, como diagnóstico diferencial o de coinfección SARS-CoV-2 y COVID-19 ^34^. Esto pudo haber impactado por aumento de la resistencia observada en algunos microorganismos debido, más no exclusivamente, al uso inapropiado de los antimicrobianos, a un incremento en su uso o a ambos factores ^35^.

En los estudios internacionales, se ha encontrado que, al comienzo de la pandemia, entre el 75 y el 95 % de los pacientes con COVID-19 recibió antibióticos a pesar de que menos del 15 % presentaba coinfección bacteriana ^36^^,^^37^. Además, el incremento del uso empírico de antibióticos en pacientes admitidos con COVID-19 grave, estuvo probablemente asociado con la neumonía asociada con el respirador y con bacteriemia, las cuales fueron reportadas como las infecciones más frecuentes ^24^. En contraste, en otros estudios se reportó que los pacientes infectados con el SARS-CoV-2 tenían tasas bajas de coinfecciones con bacterias y hongos ^38^.

Como se mencionó anteriormente, en estos estudios la disminución reportada de infecciones bacterianas y fúngicas pudo estar asociada con la suspensión de procedimientos no esenciales, sobre todo en los primeros meses de la pandemia, lo cual llevó a una disminución general del uso de los antibióticos ^39^. Infortunadamente, los estudios reportados sobre SARS-CoV-2, infecciones bacterianas secundarias y uso de antimicrobianos, no tienen datos estandarizados; utilizan diferentes definiciones, mediciones y poblaciones no comparables, lo que hace difícil establecer relaciones con certeza.

Con base en datos de los institutos nacionales de salud latinoamericanos, Thomas *et al.* reportaron un incremento en el número de enterobacterias productoras de carbapenemasas, y se observó un aumento de más del doble durante el período 2020-2021 comparado con el 2018-2019 ^40^. Para este reporte de Thomas *et al.,* se recolectaron indistintamente los primeros aislamientos de cada paciente o todos los aislamientos resistentes a carbapenémicos, según el tipo de estudio realizado en cada país ^40^. Sin embargo, esta recolección no es aleatoria y las instituciones envían solo los aislamientos resistentes, lo cual puede mostrar una resistencia que no refleja la realidad nacional. En nuestro estudio, no se logró evidenciar un aumento significativo en la resistencia basado solo en datos de WHONET.

En este análisis, llama la atención el aumento de *S. epidermidis* aislado del torrente sanguíneo, que paso del 18 % en 2018 y 2019 al 26 o al 27 % en 2020-2021. *Staphylococcus epidermidis* puede ser un contaminante en la toma de hemocultivos, pero se ha reportado su aumento global en unidades de cuidados intensivos de adultos durante la pandemia. En México, Martínez *et al.* encontraron que la sepsis era el segundo tipo de infección en la unidad de cuidados intensivos, y que *Staphylococcus* coagulasa negativo fue la causa en el 40 % de los casos, en el período de marzo a junio de 2020 ^24^. Si bien no se puede establecer ninguna asociación, la toma de hemocultivos en pacientes críticos con catéteres centrales, podría haber influido en los datos que presentamos aquí.

Finalmente, el que no se haya encontrado un aumento en la resistencia antimicrobiana, a pesar del aparente incremento en el uso empírico de antibióticos, podría deberse a varios factores asociados con el manejo de estos pacientes, como son el incremento en las medidas de control de infecciones implementadas para proteger al personal de salud, las cuales incluyen el lavado de manos, el uso de elementos de protección personal, y mayor desinfección y limpieza en el ambiente hospitalario ^8^. Es importante continuar la vigilancia epidemiológica local en los hospitales, para entender las dinámicas de la resistencia a los antimicrobianos, las cuales son de especial relevancia en los programas de optimización de uso de antimicrobianos y en la implementación de medidas para prevenir su transmisión.
